# Gut microbiome comparability between DNA extraction kits

**DOI:** 10.1136/gutjnl-2025-336648

**Published:** 2025-09-16

**Authors:** Gregory R Young, Nurulamin M. Noor, Aryan Khirwadkar, Lauren C. Beck, IBD-RESPONSE collaborators, Mohammed Tauseef Sharip, Kevin Whelan, Nick Kennedy, Jack Satsangi, Julian R Marchesi, Luke Jostins-Dean, Christopher J Stewart, Paul A. Lyons, Miles Parkes, Christopher Andrew Lamb

**Affiliations:** 1 Translational and Clinical Research Institute, Newcastle University, Newcastle upon Tyne, UK; 2 Department of Gastroenterology, Cambridge University Hospitals NHS Foundation Trust, Cambridge, United Kingdom; 3 Department of Medicine, University of Cambridge School of Clinical Medicine, Cambridge, United Kingdom; 5 Nutritional Sciences Division, King’s College London, London, UK; 6 Royal Devon and Exeter NHS Foundation Trust, Exeter, UK; 7 Translational Gastro-Intestinal Unit, Nuffield Department of Medicine, John Radcliffe Hospital, Oxford, UK; 8 Division of Digestive Diseases, Department of Metabolism, Digestion and Reproduction, St Mary’s Hospital, Imperial College London, London, UK; 9 Kennedy Institute of Rheumatology, University of Oxford, Oxford, UK; 10 Cambridge Institute for Therapeutic Immunology and Infectious Disease, Jeffrey Cheah Biomedical Centre, Cambridge, UK; 11 Department of Gastroenterology, Newcastle upon Tyne Hospitals NHS Foundation Trust, Newcastle upon Tyne, UK

The recent commentary by Manning *et al*. highlights difficulties comparing results of gastrointestinal microbiome cohort studies (1). They demonstrate how methodological heterogeneity can substantially impact data integration. Given the high inter-individual variability in the gut microbiome, findings often require replication in large cohorts to ensure relevance and validity. To address this integration and cross-validation of findings across varied cohort studies is vital.

Several studies of inflammatory bowel disease (IBD) employ prospective microbiome sampling methodologies to assess associations between the gut microbiome and disease activity (2) or response to therapy (3). Others collect samples suitable for gut microbiome analyses without protocolising microbiome-focused primary endpoints (4). Consequently, biobanked stool samples are available to assess microbiome-linked clinical endpoints. Understanding the influence of methodological variance on microbiome compositions will enable comparison across trials, reduce study duplication, increase cost-effectiveness, and ultimately improve generalisability of findings.

We quantified the influence of two commonly used DNA extraction kits: Qiagen DNEasy PowerSoil Pro (QIAGEN, DE) and FastDNA Spin Kit for Soil (MPBiomedicals, US); on microbiome compositions of N=12 stool samples from participants with IBD (clinical characteristics available in online supplementary appendix), ZymoBIOMICS Microbial Community Standards and kit reagent controls. Both kits utilise mechanical lysis steps. All stool samples had been stored in the commonly used OMNIGene•GUT preservation reagent.

We demonstrate, via metagenomic sequencing, that use of different DNA extraction kits has limited impact on gut microbiome composition when other methodological covariates are controlled. No significant difference was observed in microbial (Qiagen: Median=2.63×10^7^; IQR=2.03×10^7^-3.06×10^7^, FastPrep: Median=2.70×10^7^; IQR=2.43×10^7^-3.01×10^7^)(P=0.36)(Figure 1a) or human reads (Qiagen: Median=2.04×10^4^; IQR=2.04×10^3^–5.51×10^4^, FastPrep: Median=3.91×10^4^; IQR=4.00×10^3^7.21×10^4^)(P = 0.64)(Figure 1b), between paired sample aliquots extracted with both kits.

Likewise, we observed no significant difference in rarefied species richness (Qiagen: Median=83.5; IQR=76.8–98.5, FastPrep: Median=79.5; IQR=77.8–96.0)(P=0.73)(Figure 1c) or Shannon diversity (Qiagen: Median=3.45; IQR=3.28–3.69, FastPrep: Median=3.40; IQR=3.19-3.58)(P=0.56)(Figure 1d).

Participant microbiome individuality was highly conserved between paired samples (P=0.001, R_2_=0.97)(Figure 2a,b). Kit use had a significant impact on overall microbiome structure but described less compositional variance than any clinical covariates included in PERMANOVA analysis (P=0.001, R_2_=0.01, full results in supplementary appendix). *Fusicatenibacter saccharivorans* was the only significantly differential species between extraction kits identified using MaAsLin2 (q=0.10; P=0.0005; coef=0.049), being more abundant in Qiagen (Median=1.76; IQR=0.96–2.92) than FastPrep samples (Median=0.93; IQR=0.42–1.77)(Figure 2a,b). *F.saccharivorans* was never identified in kit reagent or Zymo community controls. It’s presence showed complete convergence between paired samples (presence N = 11; absence N = 1, full results in supplementary appendix), suggesting the species was not a kit contaminant. *F.saccharivorans* was previously depleted in patients with ulcerative colitis but not Crohn’s disease, compared to controls (7). Other key bacterial species commonly associated with IBD (*E.coli, Faecalibacerium, Roseburia, and Ruminococcus spp*. (7, 8)) were not differentially abundant between paired samples included here.

Next, we compared ZymoBIOMICS Community Standard aliquots (N=3) extracted with each kit. *A priori* knowledge of true compositions enables further appraisal of kit bias. Both kits successfully identified all taxa expected in the standards. No significant difference in compositional variance (P=0.27, R_2_=0.40) or dispersion (P=0.83) was observed between paired aliquots (Figure 2c).

Previously, differences in microbiome composition driven by extraction kit were attributed to use of (or insufficient) mechanical lysis (5, 6). Both kits tested here employ mechanical lysis, resulting in negligible differences between microbiome compositions. Identifying differential features between samples extracted with relevant kits facilitates correction during subsequent cross-validation of results from different studies.

Our results suggest that researchers should be conscious of methodological impacts on microbiome profiles but comparison between studies utilizing different DNA extraction kits is possible and represents an opportunity to cross-validate conclusions of large cohort studies.

## Supplementary Material

Online Supplementary appendix

## Figures and Tables

**Figure 1:**
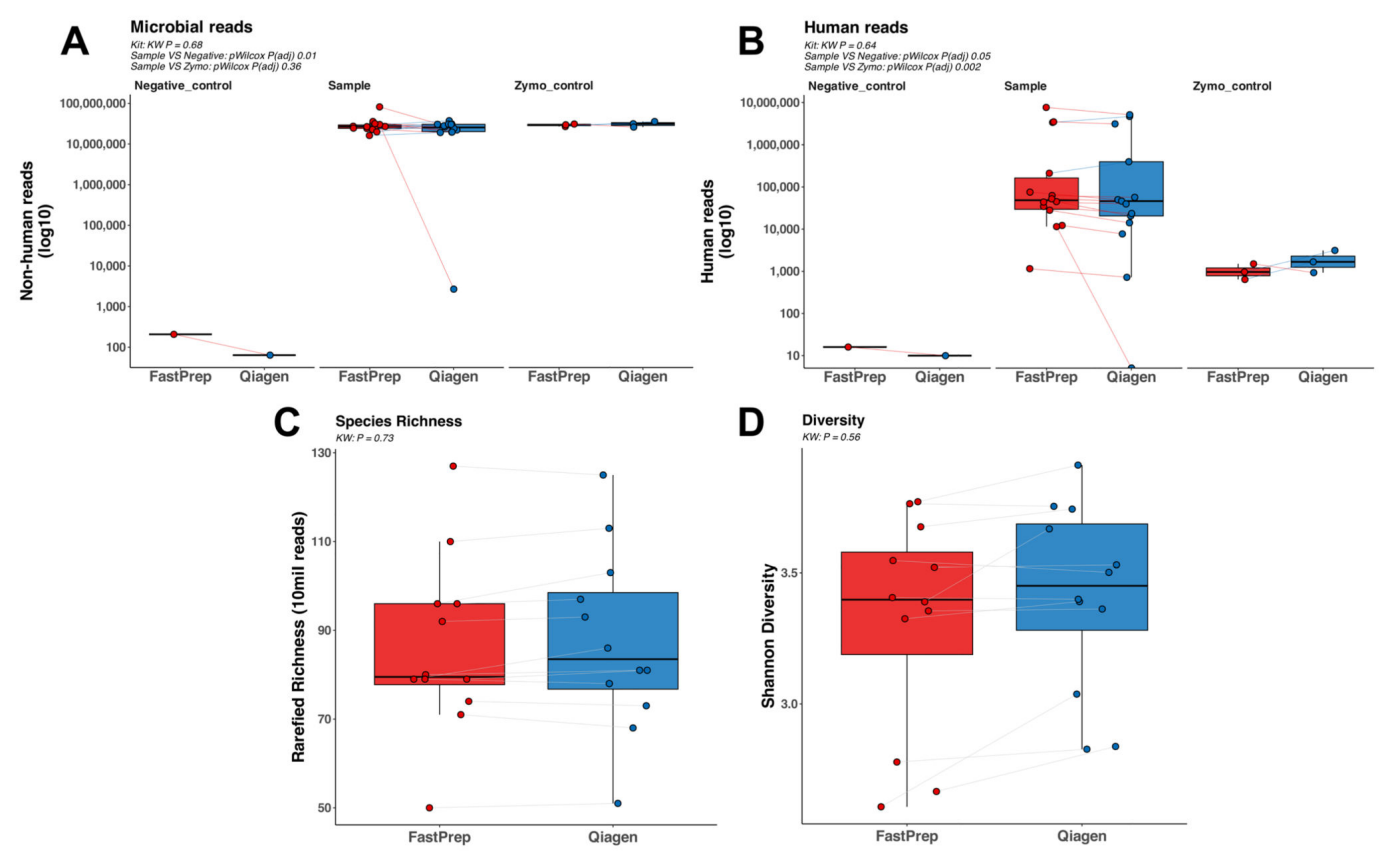
Different DNA extraction kits have no significant impact on microbial (A) or contaminating human (B) reads, nor species richness (C) or Shannon diversity (D) values in paired samples. Centre lines represent medians, boxes represent the inter-quartile range and whiskers extend to the full range of data. Each point is coloured by kit used and represents an individual sample. Grey lines connect paired samples (aliquot from the same participant stool). Summary of data used to generate plots available in online supplementary appendix. (KW = Means compared by Kruskal-Wallis rank-sum test; pWilcox P(adj) = means compared by pairwise Wilcoxon rank-sum test with Bonferroni adjustment for multiple hypothesis testing)

**Figure 2:**
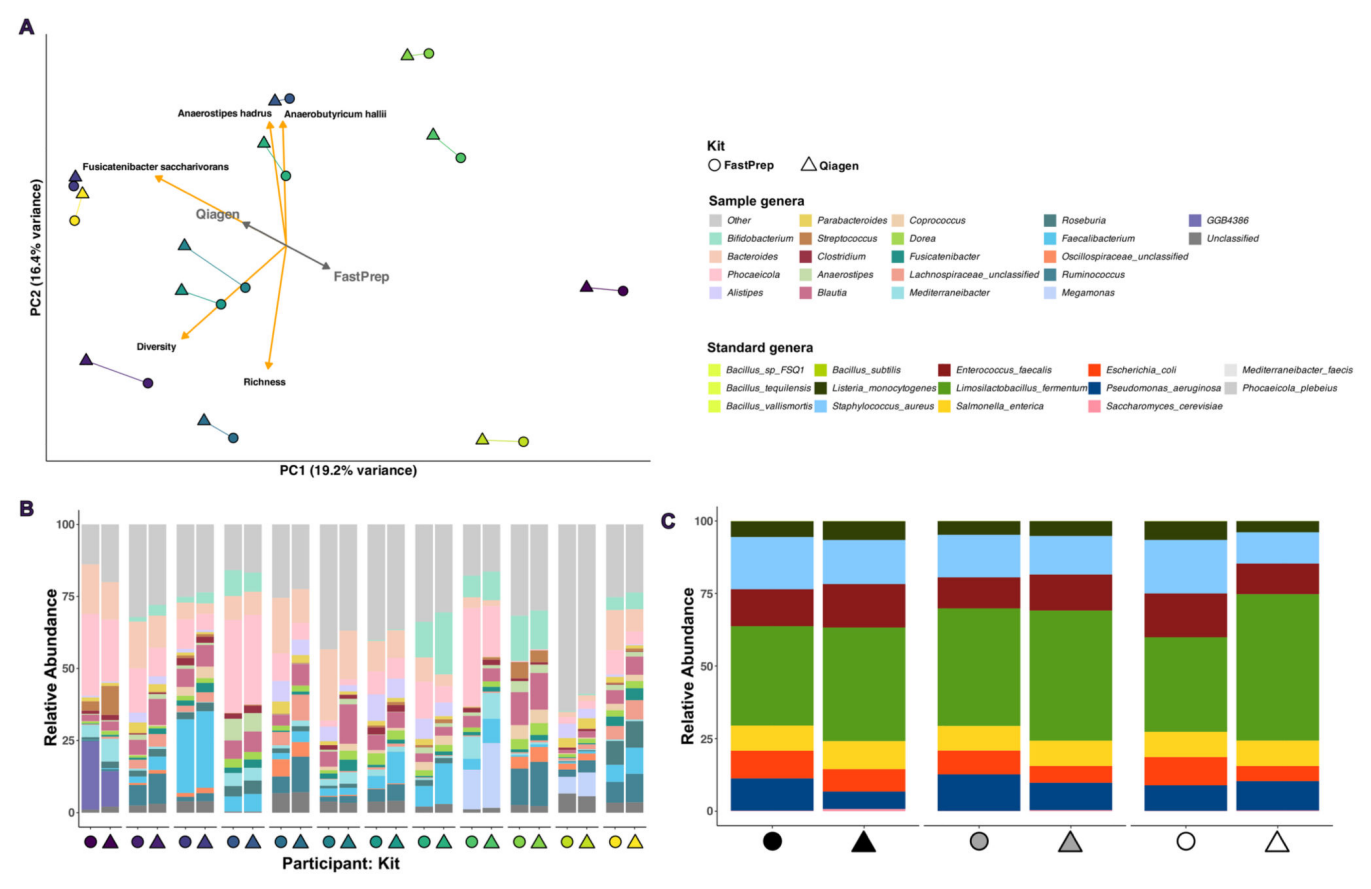
Different DNA extraction kits have no significant impact on overall community composition of paired samples from N = 12 individual participants (A,B) or paired ZymoBIOMICS Microbial Community Standards (C). Each point of the Principle Coordinates ordination based on Bray-Curtis dissimilarity is shaped by kit used (P = 0.001, R_2_ = 0.01), coloured by individual participant (P = 0.001, R_2_ = 0.97) and represents an individual sample. Factor arrows (grey) illustrate the influence of extraction kit use on ordination of points. Vector arrows (orange) illustrate the influence of microbial variables on ordination of points. Only bacterial species identified as significantly different between methodological or clinical covariates by MaAsLin2 (q < 0.25) are included as vectors. Coloured lines connect paired samples (aliquot from the same participant stool). Stacked bars labels are coloured by participant (B, as in panel A) or aliquot (C), and shaped by DNA extraction kit (as in panel A) along the x axis. Species included in barcharts represent the twenty most abundant genera across participant samples (B, accounting >50% total bacterial abundance) and all observed species in ZymoBIOMICS Microbial Community Standards (C).
