# Supplementary material for: Gut microbiome comparability between DNA extraction kits
Source: Gut. Author manuscript; Available in PMC 2026 Jun 16. (PMC7619175; doi:10.1136/gutjnl-2025-336648)
Supplement: Online Supplementary appendix [file EMS213368-supplement-Online_Supplementary_appendix.pdf]

Online Supplementary appendix

Letter: Gut microbiome comparability between DNA extraction kits

Gregory R Young, Nurulamin Noor, Aryan Khirwadkar, Lauren C Beck, IBD-RESPONSE collaborators, Mohammed T. Sharip, Julian Marchesi, Luke Jostins-Dean, Christopher J Stewart, Paul A. Lyons, Miles Parkes, Christopher A Lamb

Contents

Cohort clinical characteristics ***10***

Data summary Figure 1 (Sequencing Output) ***11***

PERMANOVA Results ***12***

Fusicatenibacter Sacchanivorans presence data ***12***

IBD-RESPONSE contributors ***13***

Cohort clinical characteristics

N = 14 participants included in sample processing.
N = 2 participants excluded due to poor sample processing.

**CATEGORICAL VARIABLES**	COUNT	PROPORTION
SAMPLES	14	-
DIAGNOSIS		
Crohn's disease	7	50%
Ulcerative colitis	7	50%
SEX AT BIRTH		
Male	5	36%
ETHNICITY		
Asian	2	14%
Black	1	7%
White	11	79%
SPECIFIC DIET		
No	2	14%
Vegetarian	12	86%
SMOKING STATUS		
Never	1	7%
Previous smoker	4	28%
Current smoker	4	28%
NA	5	36%
**CONTINUOUS VARIABLES**	MEDIAN	IQR
AGE (YEARS)	41.7	30.0 – 50.7
BMI	28.7	26.0 – 32.3
MONTHS SINCE DIAGNOSIS	64.0	13.9 – 104.0
BASELINE PRO-2 (CD)	4.1	2.2 – 4.9
BASELINE PRO-2 (UC)	4.0	3.0 – 5.0
FAECAL CALPROTECTIN AT SAMPLING	306	120 - 863

Data summary Figure 1 (Sequencing Output)

**OVERALL**	TOTAL READS	MEDIAN	IQR
RAW READS	1.00×10^9	2.79×10^7	2.45×10^7 - 3.19×10^7
QC READS	9.76×10^8	2.69×10^7	2.41×10^7 - 3.11×10^7
MICROBIAL READS	9.48×10^8	2.69×10^7	2.26×10^7 - 3.05×10^7
HUMAN READS	2.88×10^7	3.12×10^4	1.63×10^3 – 6.58×10^4
**STRATIFIED MICROBIAL READS**	TOTAL READS	MEDIAN	IQR
SAMPLE TYPE			
Samples	7.67×10^8	2.68×10^7	2.26×10^7 - 3.05×10^7
Kit negative	272	136	100 - 172
ZymoBIOMICS standard	1.81×10^8	3.05×10^7	2.76×10^7 - 3.14×10^7
EXTRACTION KIT			
Qiagen	4.38×10^8	2.63×10^7	2.03×10^7 - 3.06×10^7
FastPrep	5.10×10^8	2.70×10^7	2.43×10^7 – 3.01×10^7
**STRATIFIED HUMAN READS**	TOTAL READS	MEDIAN	IQR
SAMPLE TYPE			
Samples	2.88×10^7	4.56×10^4	1.89×10^4 – 2.58×10^5
Kit negative	26	13	12 – 15
ZymoBIOMICS standard	8.83×10^3	1.23×10^3	938 – 1.63×10^3
EXTRACTION KIT			
Qiagen	1.51×10^7	2.04×10^4	2.04×10^3 – 5.51×10^4
FastPrep	1.36×10^7	3.91×10^4	4.00×10^3 - 7.21×10^4

PERMANOVA Results

Individual patient is main driver of compositional similarity (P = 0.001, R2 = 97%).

Due to high influence of individual patient, subsequent analyses controlled for this by arranging covariates in the model formula to assess impact of kit before all other parameters, and patient last.

Formula = `adonis2(Sample_dist ~ KIT + BMI + Diagnosis + Age + Restricted_diet + PATIENT, data = Samples_META_transfer, by = 'terms')`

COVARIATE	R2	PR(>F)
Restricted diet	0.11023	0.001
Age	0.10740	0.001
BMI	0.08265	0.001
Diagnosis	0.08258	0.001
Kit	0.01039	0.001
Patient	0.58363	0.001
Residual	0.02312	
Total	1	

Fusicatenibacter Sacchanivorans presence data

F.saccharivorans was identified not identified in negative controls processed with either of the Qiagen or FastPrep kits.
F.saccharivorans was present in n = 11 samples and absent in n = 1. Where *F.saccharivorans* was identified in patient samples using one kit it was always identified in the other (n = 12).

FUSICATENIBACTER SACCHARIVORANS

KIT	Median	IQR	Prevalence (Sample #)	Absence (Sample #)	Convergence
**FASTPREP**	0.931	0.42 – 1.77	11	1	12
**QIAGEN**	1.76	0.96 – 2.92	11	1	12
**KITS**	0	0	0	2	2

F.saccharivorans was never identified in Zymo positive controls (n = 3 per kit).

IBD-RESPONSE contributors

Badr Abdalla, Natalie Agius, Tariq Ahmad, Debbie Alexander, Emma Ali, Dean Allerton, Carl Anderson, Smantha Baillie, Georgina Bates, Ian Beales, Lauren Beck, Zareen Bheekhun, Jane Biala, Jonathan Blackwell, Joanne Bone, Simon Borg-Bartolo, Louise Bowlas, Biljana Brezina, Johanne Brooks-Warburton, Amy Buckley, Helen Burton, Jeffrey Butterworth, Beatriz CAMESELLA PEREZ, Jade Chisholm, Lee Choong, Bessie Cipriano, Tonia Clark, Sonya Collins, Rachel Cooney, Jessica Cordle, Cristina Cotobal Martin, JR Cummings, Ayokunnumi Dada, Helen Dallal, Kate Datta, Jiezel De Guzman, Juan de la Revilla Negro, Aminda De Silva, Anjan Dhar, Raymoncler Diaz, Benjamin Disney, Chaonan Dong, Mary Doona, Jennifer Doyle, Dharmaraj Durai, Jill Effard, Alexander Elford, Laura Fachal, Francesco Ferraro, Mary Flowerdew, Stephen Foley, Katherine Frith, Natalia Fumis, Hannah Gibson, Zaireen Mae Gico Pios, Rachel Godfrey, Kay Goulden, Neethu Gouri Das, Marie Green, Paul Harrow, Ailsa Hart, Philip Harvey, Victoria Hildreth, Hannah Husband, Peter Irving, Katherine Jacques, Lauren John, Heather Johnson, Marie Jones, Luke Jostins-Dean, Sunita Juliao, Nicholas Kennedy, Alexandra Kent, Uzma Khan, Andrew King, Klaartje Kok, Konrad Koss, Yasmin Laidler, Christopher Lamb, Isabel Laszczak, Sarah Lawrence, Emma Lee, James Lee, Irish Lee, Charlie Lees, Robert Lees, Trevor Liddle, Jimmy Limdi, James Lindsay, Dennies Lopez, Melanie Love, Mary Lucas, Hannah Mabb, Sadek Malas, Julian Marchesi, Naomi McGregor, Simon McLaughlin, John McLaughlin, Claire McQuillian, Helen Millage, Ibinye Minimah, Matthew Morris, Alison Moss, Robert Mulligan, Elaine O'Reilly, FELICIA ONOVIRAN, Debra Owens, Christopher Palmer-Jones, Simon Panter, Ji Park, Miles Parkes, Gareth Parkes, Alina Pascau, Chirag Patel, Kamal Patel, Sally Payton, Mirela Petrova, Jahleesha Pinney, Kathryn Potts, Nick Powell, Sam Powles, Kiran Prabhu, Natalie Prescott, Aisling Quinn, Eduardo Quintero, Ajaysuraj Rachuri, Chandni Radia, Monira Rahman, Sohail Rahmany, Tim Raine, Praveen Rajasekhar, Arvind Ramadas, Krithivasan Raman, Lucia Ramirez Navarro, Parizade Raymode, Jess Record, Madeleine Richardson, Susan Ritchie, Francesca Rosa, Jack Satsangi, John Saunders, Lydia Scarlett, Shaji Sebastian, Jahnavi Seema, Christian Selinger, Krishna Shah, Anita Sharda, Mohammed Sharip, Natalie Shields, Pamela Simmons, Oliver Skipper, Melissa Smith, Ally Speight, Alan Steel, Alison Stephens, Christopher Stewart, Michelle Strickland, Sreedhar Subramanian, Alison Talbot, John Thomas, Valentina Toska, Artemis Trikola, Robert Varley, Ajay Verma, Busiswa VUYELELE TITCHMARSH, Hannah Watson, Andrea Webster, Emma Wesley, Kevin Whelan, Simone Whiteoak, Kate Wistance, Ruth Wood, Nicola Wyatt, Yifan Xu, Mark Yongco, Gregory Young, Louise-Jane Young
